# Exploration of Neurodegenerative Diseases Using Long‐Read Sequencing and Optical Genome Mapping Technologies

**DOI:** 10.1002/mds.30151

**Published:** 2025-03-03

**Authors:** Guillaume Cogan, Kensuke Daida, Cornelis Blauwendraat, Kimberley Billingsley, Alexis Brice

**Affiliations:** ^1^ Laboratory of Neurogenetics National Institute on Aging Bethesda Maryland USA; ^2^ National Institute on Aging and National Institute of Neurological Disorders and Stroke, National Institutes of Health Center for Alzheimer's and Related Dementias Bethesda Maryland USA; ^3^ Institut National de la Recherche Médicale‐U1127, Centre National de la Recherche Scientifique‐UMR7225, APHP Sorbonne Université, Institut du Cerveau—Paris Brain Institute—ICM Paris France; ^4^ Département de génétique médicale, UF de Neurogénétique Moléculaire et Cellulaire AP‐HP Sorbonne Université, Hôpital Pitié‐Salpêtrière Paris France; ^5^ Department of Neurology, Faculty of Medicine Juntendo University Tokyo Japan

**Keywords:** long‐read sequencing, optical genome mapping, neurodegenerative diseases

## Abstract

Genetic factors play a central role in neurodegenerative disorders. Over the past few decades, significant progress has been made in identifying the causative genes of numerous monogenic disorders, largely due to the widespread adoption of next‐generation sequencing (NGS) technologies in both research and clinical settings. However, many likely monogenic disorders still lack an accurate molecular diagnosis, primarily because conventional NGS methods are not effective at detecting structural variants and repeat expansions, both of which are crucial in many neurogenetic diseases. Recently, long‐read sequencing (LRS) and optical genome mapping technologies have emerged as powerful tools, offering the ability to capture more complex genetic variations. These technologies have already led to the discovery of novel genes responsible for well‐characterized neurodegenerative diseases (ND), enhancing the understanding of the biological underpinning of these conditions. Although currently LRS is mostly used in a research setting, we anticipate broader implementation of these methods in clinical laboratories in the near future. In this review, we explore the contributions of these technologies to ND research and highlight the remaining challenges for future advancements. © 2025 The Author(s). *Movement Disorders* published by Wiley Periodicals LLC on behalf of International Parkinson and Movement Disorder Society. This article has been contributed to by U.S. Government employees and their work is in the public domain in the USA.

Among neurological disorders, neurodegenerative diseases (ND) are characterized by chronic progressive loss of neuronal cells in the central and peripheral nervous systems, manifesting with a wide range of symptoms usually starting late in life. The main features are an isolated or combined presentation of motor disturbance, cognitive decline, and psychiatric disorders, ultimately leading to disability and death after a few years to decades. These diseases are often characterized by the presence of abnormally aggregated proteins, which neuropathologically define each neurodegenerative disorder. Despite ongoing efforts to improve early and accurate diagnosis, the majority of NDs still lack effective treatments to slow or halt the irreversible degeneration process.

Most ND cases result from a combination of aging, genetic, and environmental factors. However, for a subset of patients, usually with an early onset, a positive family history, or distinct atypical presentations, the disease is caused by DNA alterations in a single gene. Pathogenic variants in these genes can range from single‐nucleotide variants (SNV), which involve a change in a single nucleotide of the DNA sequence, to more complex structural variants (SV)—typically defined as changes in the DNA sequence >50 base pairs, such as deletions, duplications, inversions, translocations, and insertions. Another type of variant is repeat expansions, where a short DNA sequence is repeated in tandem, for example, the GGGGCC expansion in *C9orf72* involved in frontotemporal lobar dementia.^1^ Various modes of inheritance have been observed across NDs, whether autosomal dominant, autosomal recessive, or X‐linked.

Despite recent advances in the diagnostic yield of most NDs thanks to short‐read sequencing (SRS), many individuals with likely monogenic conditions remain undiagnosed, even after extensive genetic testing, as standard technologies continue to encounter technical limitations.^2^, ^3^

In this review, we focus on the two main emerging technologies used to address genetically undiagnosed cases, long‐read sequencing (LRS) and optical genome mapping (OGM). Emphasized by examples, we discuss their applications for NDs, the significant achievements already made, and the remaining challenges for the future.

## LRS Technologies

**FIG. 1:**
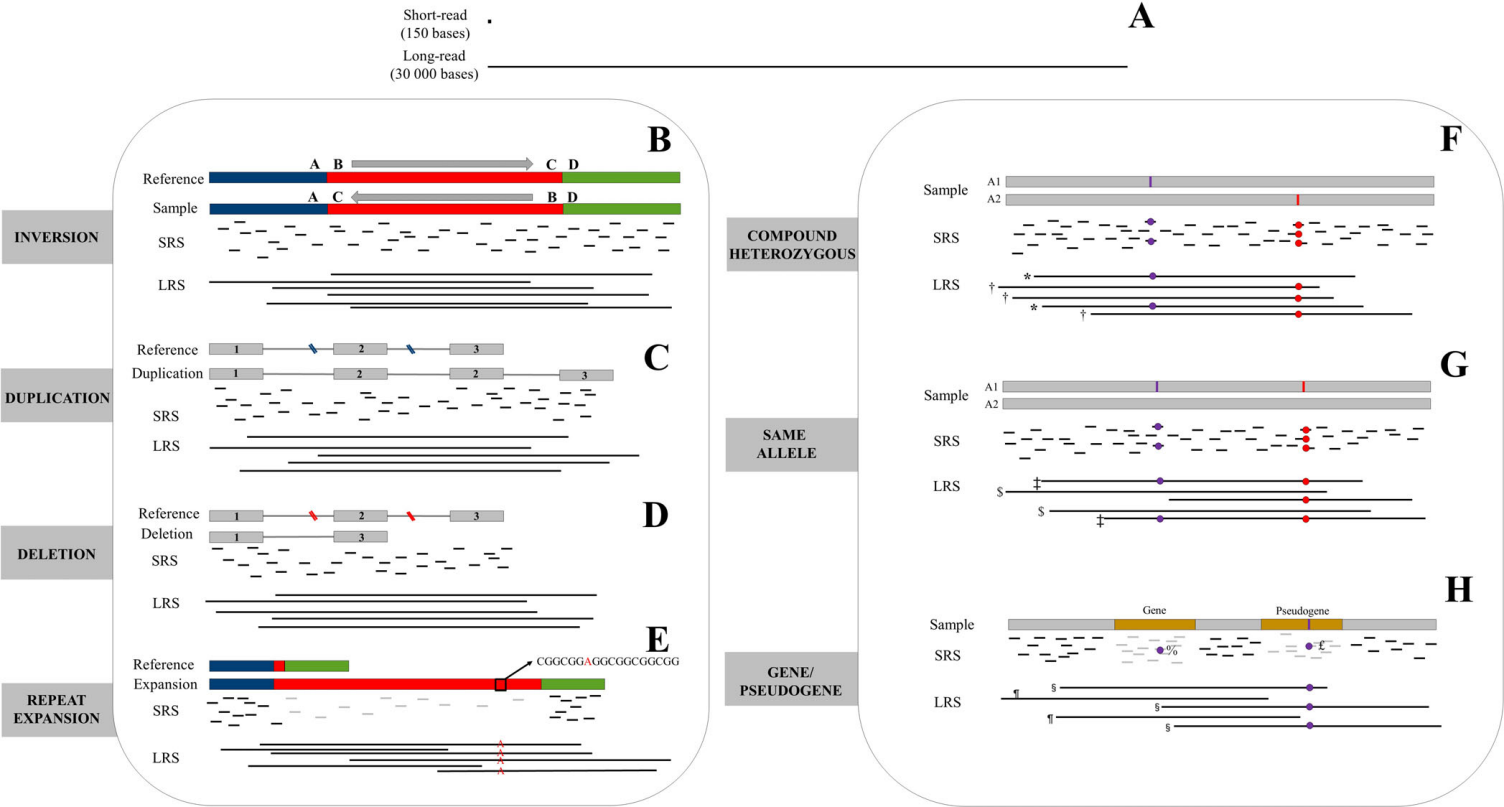
Schematic overview of the advantages of long‐read sequencing in complex variant detection. (**A**) Representation of an average short read and long read at scale (150 bp and 30 kbp). (**B**) Short reads cannot fully encompass the inverted segment (red segment), therefore likely missing the inversion in contrast with long reads. Furthermore, short reads within the rearrangement do not provide any information on the inversion, whereas most of the long reads encompass at least either the abnormal “AC” or “BD” sequences, facilitating the identification of the inversion. For copy number variations, most of the long reads identify either (**C**) two copies of exon 2 in a row for the duplication or (**D**) the absence of exon 2 (junction of exons 1 and 3) for the deletion, whereas any of the short reads would capture the rearrangement. (**E**) The red segment is expanded compared to the reference. If >150 bp, “in‐repeat” short reads would not be able to map within the expansion (gray reads), whereas long reads are either able to go through the full expansion or capture most of it. In addition, the interruption motif (the red A) is captured by long reads and not by short reads. For the phase of the variant (**F** and **G**), if the distance is longer than the length of short reads (150 bases usually), long reads can solve the phase because they encompass both variants, either showing one of the two if they are in *trans* († and *) or the two (‡) or any ($) if they are in *cis*. (**H**) The sequences of genes and pseudogenes are very similar, hampering the ability of short reads to correctly map either one (in gray), unlike long reads. The consequence is that, if a variant occurs either in the gene or the pseudogene (the purple trait in the scheme), short reads are unable to accurately assign it to the correct DNA location (% and £). Because a single long read can capture both and surrounding sequences, they accurately map the variant (¶ and §). Of note, reads shown in the schemes are not at scale. A1, allele 1; A2, allele 2; SRS, short‐read sequencing; LRS, long‐read sequencing. [Color figure can be viewed at wileyonlinelibrary.com]

Unlike SRS, which typically produces sequence reads ~50 to 300 base pairs in length, LRS produces much longer reads ranging from 1 kb to a few megabases in length (Fig. 1A).^4^ Two commercial platforms offer LRS through distinct technologies: Pacific Biosciences (PacBio) with its single‐molecule real‐time (SMRT) sequencing and Oxford Nanopore Technologies (ONT) with nanopore sequencing.^5^ The available platforms for PacBio include RS, RSII, Sequel, Sequel II, Revio, and, most recently, the Vega system. MinION, PromethION, GridION, and SmidgION are provided by ONT. Although ONT initially had a higher base‐level error rate compared to PacBio's high fidelity sequencing,^6^, ^7^ both technologies are now comparable in sequencing quality for detecting all types of genetic variations.^8^ However, ONT offers several advantages: it provides significantly longer reads (up to several megabases vs. 15–20 kb for PacBio),^9^ is more cost effective, and provides overall greater scalability for large‐scale sequencing projects at the time of writing.^7^, ^10^ A detailed overview of the distinctive features, advantages, and applications of both technologies was recently published.^11^

LRS improves variant detection by providing better mapping of complex genomic regions using longer sequence reads, leading to more accurate identification of complex variants. Its ability to map repetitive regions allows for precise detection of repeat expansions and facilitates variant phasing across complete haplotypes.^12^ LRS offers clear advantages over SRS in detecting a broader range of variants, including SNVs in difficult‐to‐map regions, indels, SVs, and repeat expansions.^4^, ^13^ Another major benefit of LRS is its capacity to detect epigenetic modifications, such as cytosine and adenine methylation, directly from sequencing data without the need for additional library preparation steps.^14^, ^15^, ^16^ LRS technologies can be used for whole‐genome sequencing or in more targeted approaches, which allow for focused examination of specific regions across many samples at a lower cost. Regions of interest can be selectively targeted, using Cas9‐based techniques (discontinued by ONT on the R9 flowcells for low‐yield reasons, therefore only available with PacBio and ONT R10 flowcells from now on)^17^ or the ONT “ReadUntil” feature, also known as adaptive sampling,^18^ which enriches coverage of regions of interest. This deeper coverage improves variant detection and increases the likelihood of identifying potential somatic variants, which are present only in certain organs, tissues, or cells of the body and exhibit lower allele frequencies.^19^ Another way to deepen the coverage is to sequence only amplicons produced by a long‐range polymerase chain reaction (PCR) to obtain reads of several kilobases.^20^, ^21^, ^22^ This approach also reduces sequencing costs by focusing on targeted regions rather than whole‐genome sequencing. However, it is important to note that long‐range PCR may introduce amplification bias, potentially leading to uneven coverage or missing certain alleles, particularly in regions with high GC content or repetitive sequences. Long‐range PCR also loses methylation information.

## Achievements of LRS in Neurodegenerative Disorders

### Identification of Complex Rearrangements Missed by SRS

The clinical features of several NDs often suggest the involvement of one or a few specific genes. This is the case for *PRKN*, which encodes Parkin, the most frequent gene implied in autosomal recessive Parkinson's disease (PD).^23^ The phenotype of *PRKN*‐PD is distinct, typically featuring an early age at onset with a median age of 32. Patients generally exhibit a strong response to levodopa treatment, experience slow disease progression, and rarely present with nonmotor symptoms such as urinary disturbances, cognitive impairment, or hallucinations.^23^ *PRKN* is localized in the FRA6E region, a genomic site known to be prone to complex rearrangement,^24^ making SVs frequently involved in its pathogenicity. Despite a clear phenotype associated with *PRKN*‐PD, the genetic cause remains unresolved in many cases. Applying LRS in twins manifesting *PRKN*‐PD with a heterozygous pathogenic variant primarily identified by SRS, we uncovered a 7.4‐Mb inversion in *trans* (one variant on each allele), an SV that had gone undetected by earlier SRS (Fig. 1B).^25^ In a similar case report, we demonstrated the utility of LRS in a family with two siblings affected by typical *PRKN*‐PD in which conventional genetic tests had identified only one copy of exon 4.^26^ LRS later revealed a more complex genetic rearrangement: a compound heterozygous 172‐kb deletion covering exons 3 and 4 and a 103‐kb duplication of exon 3 (Fig. 1C,D). The balanced rearrangement (with two copies of exon 3) and the deep intronic localization of all four breakpoints were invisible to standard methods such as multiple ligation probe amplification and exome sequencing. In total, LRS could solve a quarter of undiagnosed *PRKN*‐PD with one pathogenic variant.^27^ In the future, LRS could be used as a follow‐up strategy after identifying a heterozygous pathogenic variant in an autosomal recessive gene linked to NDs and thus provide critical genetic diagnoses back to patients.

Another example of the ability of LRS to detect SVs that were previously undetectable by conventional methods is observed in Hereditary Spastic Paraplegia (HSP), a disease characterized by bilateral spasticity, hyperreflexia, and weakness of the lower limbs.^28^ To date, 79 genes are known to cause disease. However, the diagnostic yield of SRS for HSP ranges from only 30% to 50%, depending on whether a targeted or whole‐exome sequencing approach is used.^29^ In five HSP families undiagnosed after exome sequencing, LRS identified four pathogenic *SPAST* deletions and one pathogenic deletion of *PSEN1*.^30^ *SPAST* is the most common gene associated with HSP. *PSEN1* is primarily involved in Alzheimer's Disease (AD); however, about 10% of individuals affected by *PSEN1‐*related AD show a spastic paraplegia phenotype in the early course of their disease, particularly for *PSEN1* variants located in exons 8 and 9.^31^ Collectively, these findings highlight the potential of LRS to uncover SVs and complex rearrangements that standard sequencing methods fail to detect, offering new insights into a range of NDs.

### Discovery of New Causal Genes

In addition to providing new molecular insights into genes already associated with NDs, over the past years LRS has enabled the identification of pathogenic repeat expansions in previously unknown genes associated with well‐characterized phenotypes.

Neuronal intranuclear inclusion disease (NIID) is a clinically heterogeneous disease characterized by dementia, cerebellar ataxia, peripheral neuropathy, autonomic dysfunction, and parkinsonism.^32^ The pathological hallmarks of the disease are eosinophilic hyaline intranuclear inclusions, present in various tissues such as fibroblasts.^33^ Since the disease was first described in 1968, linkage analysis had mapped the locus on chromosomes 1p.22.1‐q21.3 and 1p13.3‐23.1, but extensive molecular investigations could not decipher the molecular basis. It was only in 2019 that LRS revealed that NIID is caused by a GGC expansion in the 5′UTR region of the *NOTCH2NLC* gene (Fig. 1E).^34^ This discovery in all NIID Japanese cases was replicated in a Chinese cohort using LRS.^35^, ^36^ Despite GC‐rich expansions in the 5′UTR regions known to affect gene expression through methylation modifications, the study of methylation profiles due to LRS between NIID patients and controls showed no significant differences.^37^ Nevertheless, Deng et al revealed that the expansion of unaffected parents carrying a large expansion (>300 repeats) was hypermethylated compared to their affected offsprings with shorter expansions (148 and 172 repeats).^38^ Furthermore, they demonstrate that the expansion leads to the formation of RNA foci having a toxic gain of function by the sequestration of RNA binding proteins into p62 positive intranuclear inclusions. Therefore, they suggest that the hypermethylation of large expansions likely decreases the expression of the expanded *NOTCH2NLC* allele, hampering the formation of RNA foci and explaining why larger expansion may not be pathogenic. Another pathogenic mechanism at play for NIID is the translation of a polyglycine‐containing protein named uN2CpolyG from an alternative upstream open reading frame, which causes neuronal cell death in cell and animal models.^39^

More than 300 genes are causally related to spinocerebellar ataxia (SCA).^40^ SCA4 is an autosomal dominant ataxia first described 25 years ago, characterized by prominent axonal sensory neuropathy without ocular movement abnormalities. Multiple investigations of the locus mapped to chromosome 16q22.1 in two families within an ~8‐Mb region were inconclusive.^41^, ^42^ Using targeted LRS, four different groups recently identified a GGC repeat expansion of 48 to 57 repeats in the final coding exon of the *ZFHX3* gene in affected individuals.^43^, ^44^, ^45^, ^46^ Although this expansion could theoretically have been detected by SRS using tools like ExpansionHunter because of its size, the wide variety of potential variants made targeted LRS a more logical approach for discovering the causative variant in a variant‐agnostic manner within the targeted candidate region.

In addition to NIID and SCA4, the underpinning expansion of other NDs in which the locus had been searched for years, such as oculopharyngodistal myopathy^47^, ^48^, ^49^ and benign adult familial myoclonus epilepsy, was discovered through LRS.^50^, ^51^, ^52^, ^53^, ^54^, ^55^, ^56^, ^57^, ^58^

### Characterization of Repeat Expansions

For repeat expansions, several elements such as the size, motif, and presence or absence of an interruption are critical to characterize because they influence factors such as age of onset, penetrance, inheritance, and clinical phenotype. This is the case for the *RFC1* gene, in which biallelic pentanucleotide repeat expansion of the motifs AAGGG, AGGGC, AAGGC, AGAGG, and extremely large AAAGG (>500 repeats) in intron 2 contributes to cerebellar ataxia, neuropathy, and vestibular areflexia syndrome and less commonly a range of other NDs.^59^, ^60^, ^61^, ^62^ Importantly, expansions of other motifs such as AAAAG, AAAGGG, AAGAG, and short AAAGG are not pathogenic, which emphasizes the need for correctly assessing the motif. In a recent study, Alvarez Jerez and colleagues identified 5 individuals in a large short‐read sequenced PD cohort potentially carrying biallelic expansion in *RFC1*.^63^ However, LRS determined that one of the predicted carriers was a false positive, with an expansion of a nonpathogenic motif on one allele. After the identification of the GAA repeat expansion in the *FGF14* gene in three families using ExpansionHunter Denovo^64^ from whole‐genome SRS data,^65^ responsible for SCA27B, Pellerin and colleagues employed in the initial study targeted LRS on long‐range PCR amplicons to precisely characterize the pathogenic motif. Subsequent LRS studies observed that the presence or absence of interruptions and the content of the repeat influenced somatic variability, penetrance, age at onset, and transmission risk of SCA27B.^66^, ^67^, ^68^ Interestingly, the motif of the 5′ flanking region, which affects the stability of the repeat,^69^ would play a role in the pathogenic formation of secondary structures at the DNA and RNA levels.^68^ These studies show a good example of the relevance of LRS in characterizing repeat motifs compared to SRS.

Furthermore, interruptions within repeat expansions are of relevance in NDs, because they influence disease severity, penetrance, phenotype, and transmission risk in several neurological expansion disorders, including myotonic dystrophy type 1, SCA2, SCA10, and fragile X tremor ataxia syndrome (FXTAS).^37^ In clinical practice, these elements are of relevance because they influence genetic counseling and further individuals' reproductive decisions. For example, a CGG trinucleotide repeat in the 5′ region of the *FMR1* gene, localized on the X chromosome, causes several syndromes depending on the repeat length. Expansions >200 cause fragile X syndrome, whereas premutations spanning 55 to 200 repeat units cause FXTAS,^70^ which is characterized by gait ataxia, intention tremor, and cognitive impairment. In women, a premutation can expand to a full mutation in the next generation. However, AGG interruptions occurring every 9 to 10 CGG repeats have been shown to stabilize the repeat and reduce the risk to transmit a full expansion.^71^ Current routine genetic diagnostics for expansions (PCR‐based methods or SRS) often lack the ability to detect these interruptions, especially in GC‐rich content regions, unlike PacBio and ONT LRS (Fig. 1E).^72^, ^73^, ^74^ Leveraging the potential of LRS to tackle these disadvantages, Erdmann and colleagues recently developed an amplification‐free method to analyze a panel of 10 common ataxias repeat loci (named Clin‐CATS) using ONT Cas9‐targeted sequencing. This panel included SCA1, SCA2, SCA3, SCA6, SCA7, SCA17, SCA8, FXTAS, Friedreich's Ataxia, and *RFC1* spectrum disorders.^75^ In a cohort of 100 genetically unresolved SCA cases, this method achieved a diagnostic yield of 28%. Other research groups have explored similar amplification‐free LRS approaches targeting additional genes (eg, *HTT*, *FMR1*, *ATXN10*, and *C9orf72*).^19^ If implemented in clinical laboratories, this method has the potential to significantly improve our understanding of genotype–phenotype correlations and enhance genetic counseling for the families of affected individuals.

### Phasing and Gene–Pseudogene Distinction

In autosomal recessive disorders, when two variants are identified, it is crucial to correctly phase them and demonstrate that they are in *trans* to confirm they are the source of the disease. However, it is difficult in the clinical ND setting for two reasons: first, genetic tests are often performed separately due to the late age of onset and the unavailability of parental DNA, making it difficult to phase variants; second, the short size of reads from SRS is insufficient for phasing reads separated by >150 to 300 bases. In contrast, LRS reads can span from several kilobases to megabases of a single allele, enabling the determination of whether variants are located on the same (*cis*) or different (*trans*) alleles (Fig. 1F,G). Jin and colleagues identified two pathogenic variants impossible to phase in the *ATM* gene in 2 siblings with dystonia and tremor by SRS.^76^ The subsequent use of LRS demonstrated that these variants were in *trans* and thus were responsible for the disease.

Additionally, many genes involved in NDs have pseudogenes, which are inactive copies of genes that share high sequence similarity with the original gene. These pseudogenes can complicate variant detection with SRS because, unlike LRS, the short reads are often difficult to map accurately to either the gene or the pseudogene (Fig. 1H).^77^ This ambiguity makes it challenging to determine whether a variant is truly pathogenic, as exemplified with the autosomal recessive gene *SORD*, involved in axonal Charcot–Marie–Tooth disease type 2 and distal hereditary motor neuropathy. It shares high‐sequence homology with its pseudogene *SORD2P*.^78^ In a study by Grosz and colleagues, two pathogenic variants were identified using whole‐exome sequencing, but it was unclear whether the variants were located in *SORD* or *SORD2P*, as phasing was not possible.^79^ By employing ONT LRS, the team was able to phase the variants and confirm that the two variants were in *trans* and located in the *SORD* gene, not the *SORD2P* pseudogene. Equivalent higher performance of LRS over SRS in variants' phasing and distinction between genes and pseudogenes was confirmed for the gene *GBA1*, a gene for which certain heterozygous variants are risk factors for PD and which shares 96% sequence homology with its pseudogene *GBAP1*.^80^, ^81^, ^82^ Similarly, *NOTCH2NLC* that has four closely located pseudogenes with more than 99% sequence similarity benefits from LRS.^83^

### LRS Limitations, Challenges, and Opportunities for the Future

LRS still faces challenges in data management, storage, and analysis. Although wet lab protocols are steadily improving, extracting high‐molecular‐weight DNA and obtaining accurate fragment sizes remain labor‐intensive tasks, and efforts to harmonize these practices should be encouraged.^84^, ^85^, ^86^ After sequencing, a series of computationally intensive bioinformatic steps are necessary to produce final files that include both methylation data and variant calls. A number of complex genomic regions, such as homopolymers or GC‐rich repeat regions, remain difficult to sequence, especially for PacBio's polymerase. Although new standardized methods are starting to emerge,^7^ the field has yet to establish a definitive gold standard pipeline for small variants and SVs calling in LRS, in contrast to the well‐established protocols already in place for SRS. Advanced bioinformatics expertise is often necessary, though tools from ONT, PacBio, and the broader research community are becoming more accessible to nonexperienced users.^87^

**FIG. 2:**
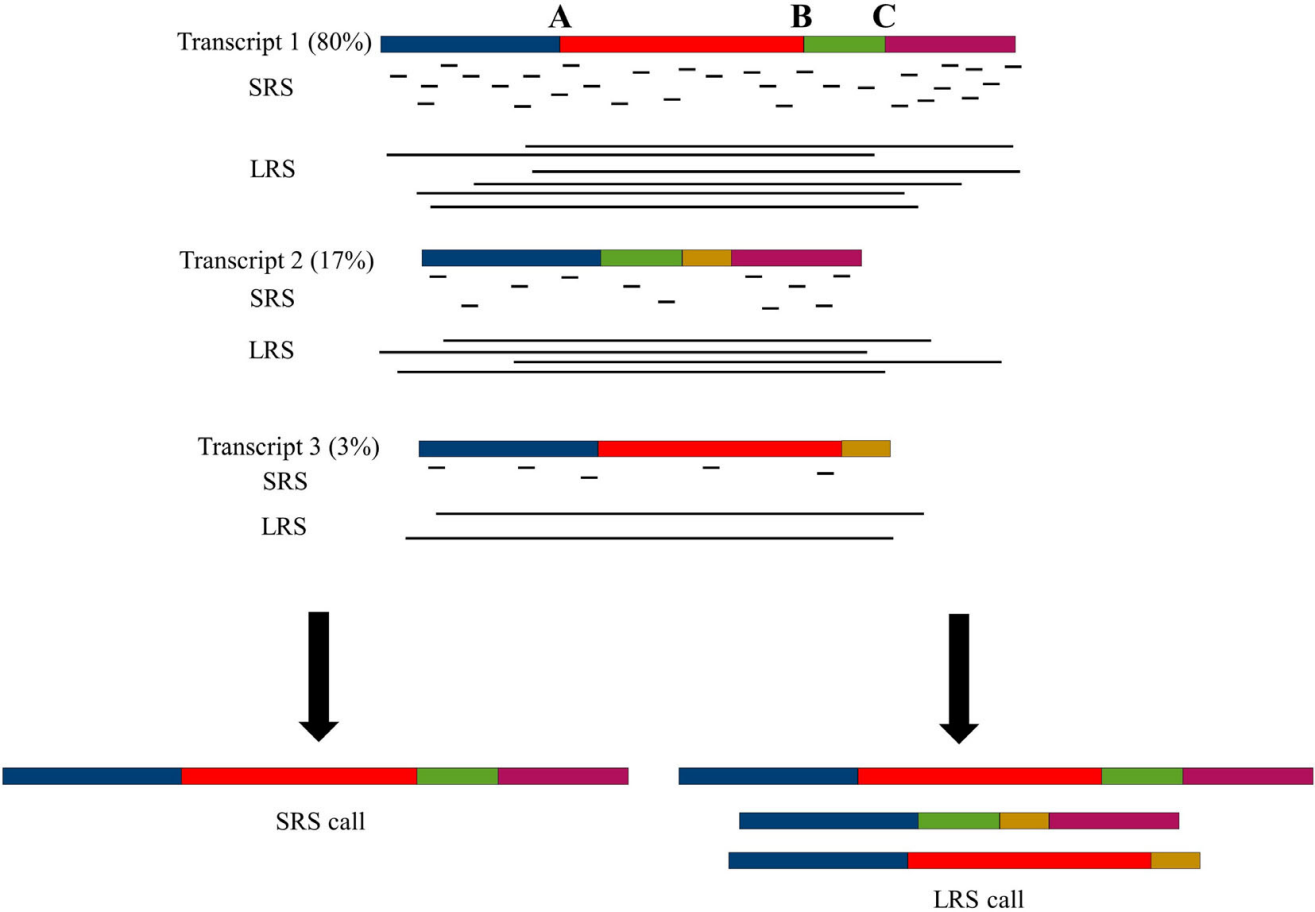
Advantages of long‐read sequencing over short‐read sequencing for transcript identification. Three artificial transcripts and their frequencies of a given tissue are exhibited along with the mapping of short‐ and long reads. The number of reads decreases by the frequency of the transcript. Colors represent exons. The yellow cryptic exon (present in minor transcripts 2 and 3 only) may not be captured by short reads, whereas long reads would identify it. Therefore, the reconstruction step required for short‐read RNA sequencing misses the full transcript landscape unlike long‐read calling, which does not require a reconstruction step. [Color figure can be viewed at wileyonlinelibrary.com]

However, several promising approaches are worth noting. The integration of methylation analysis in LRS pipelines could improve genetic diagnoses in the future, as methylation has already been shown to enhance diagnostic rates. Indeed, methylation profiling can reclassify variants of unknown significance in genes with known epigenetic signatures, such as *CREBBP* in Rubinstein–Taybi syndrome and *NIPBL* in Cornelia De Lange syndrome.^88^ It can also detect disease‐specific methylation changes caused by repeat expansions, as seen in *NOTCH2NLC* (NIID)^38^ and *FMR1* (fragile X tremor with ataxia syndrome and fragile X syndrome).^37^, ^89^ In cases where sequencing fails to identify a causal variant, abnormal hypermethylation or hypomethylation near critical genes can help prioritize regions of interest. Finally, integrating methylation profiles with RNA sequencing enables the detection of functional consequences, such as gene silencing due to promoter hypermethylation. Together, these approaches demonstrate how combining methylation analysis with LRS enhances the ability to resolve genetic diagnoses. In addition, the integration of RNA sequencing data with LRS to identify differentially expressed genes and exons will help to elucidate the impact of variants in deep intronic or regulatory regions.^90^ Indeed, SRS cannot sequence full‐length transcripts and requires reconstruction methods (Fig. 2).^91^ As a result, the current annotation of human transcript isoforms is estimated to cover only 33% of true isoforms, leaving two‐thirds unaccounted for.^92^ Furthermore, the protein model produced from these reconstructions is often incomplete. In contrast, long‐read RNA sequencing offers significant value by capturing full‐length transcripts, allowing for the detection of alternative splicing, isoform diversity, cryptic exons, novel transcripts, and new protein isoforms,^93^, ^94^ ultimately unveiling new disease pathways and potential therapeutic targets (Fig. 2). This potential to resolve complex transcriptomic alterations is underscored in a study by Alvarez Jerez and colleagues who uncovered how an intronic *GBA1* variant, which is associated with PD in African‐ancestry populations, alters splicing and reduces enzyme activity using long‐read RNA sequencing, revealing key disease mechanisms previously missed by traditional methods.^95^, ^96^ Additionally, alterations in mitochondrial DNA (mtDNA) have been reported in a number of NDs.^97^, ^98^ However, exploration of mtDNA is difficult with short reads: aligners struggle to map specific mtDNA sequences such as the control region or nuclear mitochondrial DNA segments (NUMTS, ie, mtDNA sequences localized within the nuclear genome), resulting in removal of reads and therefore poor coverage.^99^ Heteroplasmy (ie, the variability of mtDNA allele frequency depending on the cell or tissue) is also difficult to capture using SRS. LRS partially overcomes these disadvantages because a single read can easily encompass the full mtDNA sequence (~16.6 kb long), allowing for de novo assembly and detection of heteroplasmic large‐scale rearrangements. Furthermore, the inclusion of the adjacent nuclear sequences of NUMTs improves correct mapping. Although a detailed review of LRS applications for mtDNA is beyond the scope of this review, it is worth mentioning that its use has already proven useful in the context of PD.^100^

Finally, although costs have decreased, LRS remains too expensive for widespread clinical use across all NDs at this time. Barcoding, which enables the processing of multiple samples on a single flowcell, may help reduce costs in the near future. Another promising technique is OGM, which offers an alternative to LRS and typically is more automated, affordable, and addresses some of the limitations of SRS.

## Optical Genome Mapping

Historically, the detection of SVs relied on cytogenetic tools such as karyotype, fluorescence in situ hybridization, and copy number variant (CNV) microarrays (CMA).^101^, ^102^ Although these methods remain the routine genetic tests for SV detection, they come with notable limitations. Karyotyping offers a low resolution (5–10 Mb on average) and requires significant expertise, making it uncommon in neurogenetic studies. CMA provides enhanced resolution, enabling the detection of relevant microdeletions and microduplications, leading to its recommendation as a first‐tier test for developmental disorders.^103^, ^104^ However, CMA has limitations as well: it lacks sensitivity for detecting low‐level mosaicism, cannot identify balanced chromosomal aberrations such as inversions and translocations, and does not provide information on the orientation or location of insertions. Additionally, its resolution is incomplete (no detection of SVs below 20 kb). OGM offers a solution to several of these challenges.

OGM was first described in 1993.^105^ It involves imaging long, linear, single DNA molecules (>150 kb) labeled at specific sites. After enzymatic labeling at canonical sequences on megabase‐sized DNA molecules, fluorescent makers are applied.^106^, ^107^, ^108^ Using microfluidics, high‐resolution microscopy, and automated image analysis, OGM enables high‐throughput whole‐genome imaging and de novo assembly.^107^, ^109^ The technique, developed by Bionano Genomics (San Diego, California, USA), compares the fluorescent label patterns of the sample with a reference genome. Its key advantages over existing methods is the ability to detect SVs as small as 500 nucleotides, balance SVs, and precisely localize breakpoints. On average, OGM detects ~5700 SVs and 11 CNVs per sample.^108^ A reference dataset of SVs derived from 204 human population control samples is available, allowing for the effect prioritization of relevant rare variants, with a median of 80 rare SVs identified per sample.^108^, ^110^ In a proof‐of‐principle study involving 85 individuals with clinically relevant CNVs and SVs previously identified through standard cytogenetic testing, Mantere and colleagues demonstrated 100% concordance with OGM. This concordance was observed across a range of aberrations, including aneuploidies, deletions, duplications, translocations, inversions, insertions, isochromosomes, and ring chromosomes, reinforcing confidence in the sensitivity of OGM.^108^

Although OGM has been less explored than LRS in the field of NDs, it has already shown potential benefits in identifying both SVs and repeat expansions over SRS. Current methods used in clinical laboratories, such as flanking PCR and repeat‐primed PCR, often fail to fully amplify long repeats, particularly those with high GC content. The gold standard technique for repeat sizing, Southern blot (SB), is not routinely used because it is time consuming and labor intensive, and requires specialized laboratory equipment. Several studies have begun investigating whether OGM can overcome these limitations in the context of NDs.^111^, ^112^, ^113^

### Identification of Repeat Expansions

Facchini et al recently compared the performance of OGM and SB in 17 individuals with previously confirmed biallelic pathogenic expansions of more than 250 repeat units in the *RFC1* gene.^114^ The study demonstrated a strong linear correlation between OGM and SB in assessing repeat size. However, SB tended to overestimate the size of longer repeats, particularly those exceeding 1000 units. Overall, OGM provided more accurate results in approximately 25% of cases. For example, three individuals who appeared homozygous based on SB were found to be compound heterozygous with OGM, and one individual classified as compound heterozygous by SB was reclassified as homozygous using OGM. Interestingly, the authors also performed LRS on two individuals and found that, in both cases, the LRS data were more consistent with the OGM estimates than with those from SB. They concluded that, for a targeted approach focusing on repeat size only, with similar costs, OGM is easier to use and faster to perform than SB (two compared to four days), and offers a more automated workflow than LRS. As a result, OGM could potentially replace standard methods for characterizing the size of *RFC1* and other repeat expansions associated with NDs in the future. However, it is important to note that although most DNA extraction methods are compatible with SB, OGM requires highly purified, ultra‐high‐molecular‐weight DNA, which demands a more specialized extraction process. Additionally, OGM requires specific imaging equipment.

### Identification of Structural Variants

Another advantage of OGM over SRS concerns balanced genomic rearrangements, such as translocations and inversions, that are pathogenic when they disrupt a gene involved in a monogenic condition. Interestingly, in 40% of translocation cases, the breakpoints are not clear‐cut but involve one or several SVs.^115^ The ability to precisely understand the cause of the disease in the case of translocations is therefore hampered by the limited ability of SRS to capture complex SVs. Sund et al explored two unrelated individuals presenting neurological syndromes with a translocation first revealed by a karyotype.^116^ The subsequent use of Sanger and SRS to identify the gene(s) implied was not successful. Authors then used LRS and OGM, which revealed complex rearrangements at both breakpoints involving several SVs (inversions and insertions) and disrupting the sequence of two genes, both known to contribute to the same phenotype observed in the patients. In another study, Sanger sequencing could not confirm the breakpoints of an individual having Segawa disease due to an inversion disrupting *GCH1* identified by SRS.^117^, ^118^ Only the use of OGM coupled with targeted LRS deciphered a complex rearrangement at breakpoint sites.

Beyond balanced rearrangements, it is known that the detection of large SVs (ie, >50 kb) by LRS callers is limited.^119^ Using both OGM and LRS to identify SVs in two individuals with a known *SNCA* triplication (1.6 Mb) and duplication (0.3 Mb) in induced pluripotent stem cells, only OGM was able to call the large SVs.^120^ Of note, both SVs were visible by the precise analysis of mapped long reads using Integrative Genomic Viewer. Therefore, it is important to directly look at the LRS “raw” data when a specific gene is suspected from a typical phenotype, even when no variant was called by LRS callers. This study also demonstrates that, although LRS remains the method of choice for phasing because of base pair information, OGM has the possibility to phase SVs when they are very close (~1 Mb maximum) to each other (shown in two *PRKN* previously known deletions of exon 2 and exons 3 to 5 in induced pluripotent stem cells).^120^

### Limitations and Advantages of OGM

It is important to note that OGM has several limitations. First, it does not provide sequence‐level information, so neither motif nor SNV can be identified, the methylation information cannot be obtained, and its minimum sequence resolution is limited to 500 bp (Table 1). Furthermore, depending on the position of the probes, repeat expansions located between two probes may not be captured by OGM. However, OGM offers several advantages over SRS and LRS.^108^ Although interpreting SVs remains a significant challenge across all platforms, OGM's ability to accurately call large SVs with a lower false‐positive rate makes it a robust tool for validating large variants of interest detected using LRS. This capability enables a complementary and orthogonal assessment of SVs. In addition, OGM uses ultra‐high‐molecular‐weight DNA, enabling N50 values up to 250 kbp, significantly longer than the ~15 to 100 kbp typically achieved with standard LRS. This long‐range information is particularly useful for de novo assembly in challenging genomic regions, such as repetitive sequences and complex SVs. These capabilities make OGM highly effective for detecting and validating large SVs. However, the absence of single‐nucleotide resolution with OGM hampers the use of smaller variants for phasing and the ability to link SNVs to SVs. In contrast, ultra‐long LRS can generate reads exceeding 1 Mbp, enabling superior phasing across entire haplotype blocks. This includes highly repetitive regions, such as centromeres and telomeres, which are difficult to resolve using other technologies. Whereas OGM provides unique advantages for determining large‐scale structural features, ultra‐long LRS is ultimately superior for global phasing due to its ability to accurately link variants across extensive genomic regions. Nonetheless, OGM does not require library preparation as it works with native DNA molecules. It also has lower data storage requirements, does not need Graphic Processor Units (GPU) or advanced bioinformatics expertise, and can be more cost effective than LRS. Finally, OGM could replace the classical Single Nucleotide Polymorphism‐array used in clinical laboratories to identify SVs, repeat expansions, and precisely the molecular underpinnings of balanced events such as inversions and/or translocations in the future.

**TABLE 1:** Comparison of the value of SRS, LRS, and OGM

		SRS	LRS		OGM
Variant detection			PacBio	ONT	
Reads range		75–300 bp	15–20 kbp	10 kbp −1 Mbp	NA
SNVs		+++	++	++	−
SVs		+	+++	+++	+++^a^
Repeat expansions	Length	+	+++	+++	+++
	Motif	+	+++	+++	−
Methylation		++^b^	+++	+++	−
Organizational aspects					
DNA quality required		+	++	++	+++
Cost		+	+++	+++	+
Scalability		+++	+	++	+++
Storage capacity required		+	+++	+++	+
CPUs and GPUs required		+	++	+++	+

Abbreviations: SRS, short‐read sequencing; LRS, long‐read sequencing; OGM, optical genome mapping; NA, not applicable; SNVs, single‐nucleotide variants; SVs, structural variants; CPUs, central processor units; GPUs, graphic processor units.

^a^ Only more than 500 nucleotides.

^b^ Requires a specific library preparation.

### Detection of Somatic Events

A somatic variant is a genetic alteration that occurs in non‐germline cells and is acquired during an individual's lifetime, often presenting at low allele frequencies and can be cell type specific, making them difficult to detect using standard sequencing methods. OGM can effectively detect genome‐wide somatic variants due to its high depth of coverage, ranging from 300X to 1600X, whereas standard whole‐genome LRS typically reaches only about 30X coverage. This lower coverage, combined with the limitations of current long‐read repeat calling methods, can yield false positives in somatic variant analysis, particularly in repetitive or low‐complexity regions. In addition, certain somatic variants may not be captured by a relatively low number of reads, leading to false negatives. However, using targeted LRS approaches such as CRISPR‐Cas9‐targeted enrichment or long‐range PCR‐based amplicons can be efficient in detecting somatic variations of repeat expansions in NDs. For example, in X‐linked dystonia parkinsonism, a disease due to a CCCTCT repeat expansion in a SINE‐VNTR‐Alu region, Reyes and colleagues have shown that brain tissues (basal ganglia and cerebellum) present a significantly higher median repeat length and higher degrees of repeat instability than blood.^22^ Furthermore, using LRS from PCR amplicons, they were able to reveal the existence of common divergent interruptions, which affect both motif length and sequence, and therein the age at onset.^20^ In a follow‐up study, they showed that the frequency (ie, level of mosaicism) of certain interruptions in blood cells from X‐linked dystonia‐parkinsonism cases influences the stability of the repeat across generations (ie, the probability for the expansion to either expand, remain stable, or contract during parent–offspring transmission).^21^ Interestingly, Pellerin and colleagues also used LRS in various types of tissues in the context of SCA27B linked to *FGF14* GAA‐TTC expansion.^67^ They observed almost no somatic instability on peripheral and postmortem brain tissues except for the cerebellum, which showed a significantly high somatic instability. Importantly, this instability was correlated with the tissue expression and repeat length, explaining why *FGF14* repeat expansions result in a pure cerebellar phenotype. Additionally, whereas familial adult myoclonic epilepsy 2 (FAME2) and FAME3 expansions were initially detected using short‐read WGS and repeat‐primed PCR, SMRT and ONT sequencing uncovered somatic variability in repeat lengths, offering in‐depth insights into the phenotypic variability of these conditions.^57^, ^58^

## Conclusions

Despite significant advancements in LRS, particularly in neurogenetics, several challenges continue to restrict its broader clinical application. As with many rapidly evolving technologies, LRS faces issues with stability due to frequent updates in software, kit chemistries, and base‐calling methods. Additionally, unlike SRS, which benefits from extensive standardized datasets for variant comparison and allele frequency analysis, LRS lacks well‐established, comprehensive variant catalogs. However, large‐scale efforts such as the 1000 Genomes Project, the NIH's All of Us initiative, and the Center for Alzheimer's and Related Dementias initiative are beginning to address these gaps by constructing SV databases and refining variant interpretation tools to better identify causal variants.

In response to the need for greater reliability and standardization, ONT has launched the Q‐Line initiative, which is specifically designed to streamline LRS for clinical use. The Q‐Line framework aims to enhance data quality, reproducibility, and regulatory compliance by providing controlled reagent kits, optimized software, and more rigorous validation processes. This initiative represents a significant step forward in making LRS a more reliable and robust tool for clinical applications, paving the way for its broader use in diagnostic laboratories and therapeutic development. Nonetheless, for the immediate future, SRS is expected to remain the dominant technology in clinical laboratories due to its scalability, cost effectiveness, and established infrastructure. However, recent research demonstrates the unique value of LRS in discovering novel genes and determining undiagnosed cases that involve complex rearrangements or large repeat expansions. Therefore, after a negative SRS in the diagnostic procedure of patients suspected to have a monogenic cause, LRS appears to be a relevant strategy. Over time, LRS may become an all‐encompassing genetic testing approach, capable of identifying SNVs, SVs, and epigenetic modifications, thereby solidifying its place in clinical diagnostics.

## Author Roles

(1) Research project: A. Conception, B. Organization, C. Execution; (2) Statistical analysis: A. Design, B. Execution, C. Review and critique; (3) Manuscript: A. Writing of the first draft, B. Review and critique.

G.C.: 1A, 1C, 2A

K.D: 1A, 3B

C.B: 1A, 3B

K.B: 1A, 3A, 3B

A.B: 1A, 3B

## Full financial disclosures of all authors for the previous 12 months

G.C. is supported by the Global Parkinson's Genetics Program (GP2). GP2 is funded by the Aligning Science Across Parkinson's (ASAP) initiative and implemented by The Michael J. Fox Foundation for Parkinson's Research (https://gp2.org). For a complete list of GP2 members, see https://gp2.org. K.D reports receiving grants from the JSPS Research Fellowship for Japanese Biomedical and Behavioral Researchers at NIH.

## Data Availability

Data sharing not applicable to this article as no datasets were generated or analysed during the current study.
